# Three-dimensional analysis of the physiologic drift of adjacent teeth following maxillary first premolar extractions

**DOI:** 10.1038/s41598-019-51057-4

**Published:** 2019-10-10

**Authors:** Fei Teng, Fei-Yu Du, Hui-Zhong Chen, Ruo-Ping Jiang, Tian-Min Xu

**Affiliations:** Department of Orthodontics, Peking University School and Hospital of Stomatology, 22# Zhongguancun South Avenue, Haidian District, Beijing, P.R. China

**Keywords:** Oral diseases, Software

## Abstract

We assessed the three-dimensional (3D) pattern of the physiologic drift of the remaining adjacent teeth after premolar extraction due to orthodontic reasons and the associated factors. Data were collected from 45 patients aged 17.04 ± 5.14 years who were scheduled to receive a fixed appliance after maxillary premolar extraction. Seventy-five drift models were obtained and digitalized via 3D scanning. The average physiologic drift duration was 81.66 ± 70.03 days. Angular and linear changes in the first molars, second premolars, and canines were measured using the 3D method. All the examined teeth had tipped and moved towards the extraction space, leading to space decreases. Posterior teeth primarily exhibited significant mesial tipping and displacement, without rotation or vertical changes. All canine variables changed, including distal inward rotation and extrusion. The physiologic drift tended to slow over time. Age had a limited negative effect on the mesial drift of posterior teeth, whereas crowding had a limited positive effect on canine drift. Thus, the mesial drift of molars after premolar extraction may lead to molar anchorage loss, particularly among younger patients. The pattern of the physiologic drift of maxillary canines can help relieve crowding and facilitate labially ectopic canine alignment, whereas canine drift is accelerated by more severe crowding.

## Introduction

Based on the theories of Weinstein^1^ and Proffit^2^, the relative stability of tooth position is maintained by the equilibrium of intrinsic forces from the lip and tongue, extrinsic forces from habits or orthodontic appliances, dental occlusal forces, and forces from the periodontal membrane. When teeth are missing due to caries, periodontal disease, trauma, or extraction, the equilibrium will be broken, and the remaining dentition will spontaneously move to establish a new equilibrium.

Extraction has been a very common strategy in orthodontic treatment since Tweed^3^ and Begg^4^ reported it over 60 years ago. The most commonly extracted teeth for orthodontic purposes are the first premolars^5^. Although controversial^6,7^, extraction is commonly used to correct the tooth discrepancy^8^, affect the vertical dimension^9^, retract incisors^10^ and relieve crowding^11^. After extraction, the missing premolars can induce a spontaneous adjustment of dentition.

Bourdet^12^ termed this adjustment “physiologic drift”. Because this is a common phenomenon in cases with missing teeth, several animal experiments have been performed since the 1960s to confirm the drift^13–25^, establish an experimental animal model^14,21,23^, and explore the mechanism^15–20,24,25^.

Physiologic drift is important in orthodontic treatment. Some orthodontic techniques use the drift to simplify the treatment, including the methods of Alexander^26^ and PASS^27^. However, the benefit of long-term physiologic drift—including the spontaneous and uncontrolled movement of dentition—with current treatments need to be evaluated.

Previous clinical research has primarily focused on mandibular dentition^28–33^. After the extraction of lower premolars, the researchers observed mesial movement of the first molar and the distal movement of the canines, including tipping and translation. Extraction sites also tend to close, and the incisors spontaneously migrate to reduce crowding. Hence, studies have proposed a delay in the bonding of the appliances for the lower arch to take advantage of the physiologic drift. The first molar is usually the most important anchorage tooth during orthodontic treatment^34^, and its mesial movement could contribute to a potential risk of losing anchorage. However, studies^35,36^ have found that the movements of these lower first molars are minimal and only account for a small proportion of closure of the premolar extraction space during the drift.

Physiologic drift does not only occur in the lower arch^35,37–42^, but only a few studies^35,38,40,42^ have assessed the physiologic drift of maxillary dentition, and even fewer have addressed this issue in orthodontics. The maxillary arch usually requires stronger anchorage^34^ for orthodontic treatment. Thus, identification of the characteristics of physiologic drift of the upper arch following tooth extraction may help to control upper anchorage in the duration between extraction and bonding application, or even during the early stage of treatment using very soft wires. Hence, it is vital to design an appropriate research study to assess the problem.

Most previous studies^28–33,35–37^ have used manual measurement with lateral cephalometric radiographs and dental cast models to evaluate dentition drift after premolar extraction. However, accurate relative three-dimensional (3D) tooth displacement during physiologic drift has not been evaluated. In the present study, we aimed to quantify the physiologic drift of untreated adjacent teeth following first premolar extraction during the permanent dentition stage using 3D digital technology. To our knowledge, this is the first study to assess the 3D pattern of the physiologic drift of the maxillary remaining adjacent teeth after first premolar extraction for orthodontic reasons and the associated factors.

## Results

### Patient and sample cohort

The descriptive data on the characteristics of the 45 participants were as follows:

1. Sex: 10 male and 35 female;
2. Age: 17.04 ± 5.14 years, from 12 to 29 years, and the 95% confidence interval was 15.97 to 18.12 years;
3. Angle Classification^43^: Angle I, 16; Angle II, 13; Angle III, 6;
4. Crowding prior to extraction: 3.69 ± 2.41 mm, from 0 to 12 mm, and the 95% confidence interval was 2.96 to 4.41 mm.

The descriptive data on the characteristics of the 150 extraction sides at T_X_ were as follows:

1. Sex: 30 male and 120 female;
2. Age: 15.79 ± 4.49 years, from 12 to 29 years, and the 95% confidence interval was 15.06 to 16.51 years;
3. Angle Classification^43^: Angle I, 62; Angle II, 50; Angle III, 38;
4. Crowding prior to extraction: 1.79 ± 1.64 mm, from 0 to 6 mm, and the 95% confidence interval was 1.52 to 2.05 mm.
5. The average drift duration was 81.66 ± 70.03 days.
6. Distribution after grouping based on the months of drift at Tx: 1 month, 50; 2 months, 23; 3 months, 24; 4 months, 11; 5 months, 10; 6 months, 11; more than 6 months, 19.

### Correlation of the change in the physiologic drift variables with drift time

Table 1 shows the correlation between the changes in some variables and physiologic drift duration after first maxillary premolar extraction, indicating that these variables may markedly change with time (Pearson’s r, p < 0.05). Such variables included U6a, U6i, U6m, U5a, U5m, SC, and all canine variables. These variables need to be further analyzed. By contrast, changes in U6r, U6v, U5i, U5r, and U5v were not marked over time; hence, they were not assessed further.

**Table 1:** Pearson’s r between drift duration (days) and changes in the variables.

Pearson’s r	Variables											
	Angulation: mesial tipping (+) distal tipping (−)		Inclination: labioversion (+) linguoversion (−)		Rotation: mesial inward (+) distal inward (−)		Mediodistal displacement: mesial movement (+) distal movement (−)		Vertical displacement: extrusion (+) intrusion (−)		Extraction space closure: increase (+) decrease (−)	
	r	p value	r	p value	r	p value	r	p value	r	p value	r	p value
U6	0.647	<0.001	−0.414	<0.001	0.021	0.796	0.768	<0.001	0.020	0.813		
U5	0.539	<0.001	0.035	0.675	−0.080	0.328	0.756	<0.001	0.035	0.675		
U3	−0.610	<0.001	−0.441	<0.001	−0.643	<0.001	−0.662	<0.001	0.574	<0.001		
											−0.759	<0.001

U6 for the first molar, U5 for the second premolar, U3 for the canine.

### Changes in the variables of the adjacent tooth

**Figure 1:**
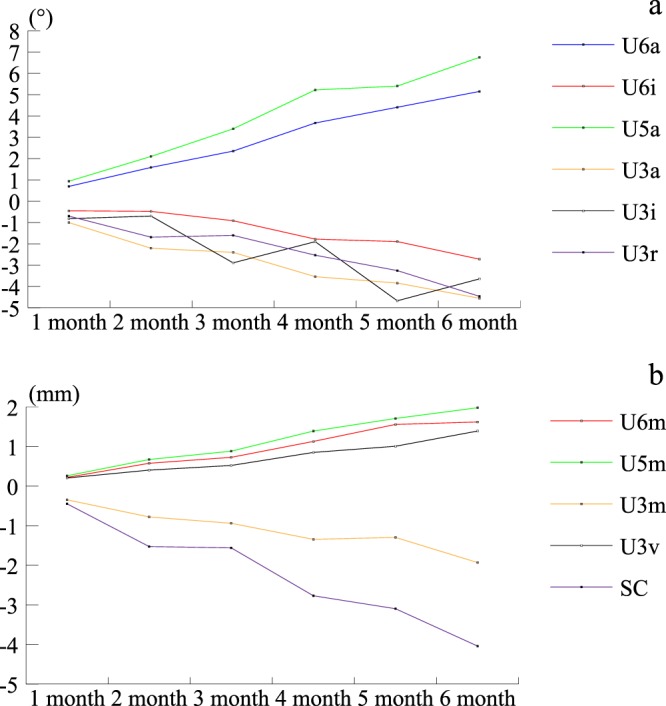
Monthly average changes in the variables of the adjacent tooth: (**a**) Changes in the angular variables. (**b**) Changes in the linear variables. Angulation: mesial tipping (+) or distal tipping (−). Inclination: labioversion (+) or linguoversion (−). Rotation: mesial inward rotation (+) or distal inward rotation (−). Mesiodistal displacement: mesial movement (+) or distal movement (−). Vertical displacement: extrusion (+) or intrusion (−). Extraction space closure: space increase (+) or space decrease (−).

The abovementioned markedly changed variables were selected, and the average monthly change was estimated and depicted using line charts (Fig. 1). Only a few cases had extraction sides with drifts over 6 months; hence, only the data from the first 6 months were considered when assessing the extent of the changes.

U3 markedly changed in all directions. U3 inclined lingually and distally, and showed distal inward rotation, extrusion, and distal movement. U6 and U5 had tipped and displaced mesially, and U6 also showed changes in lingual inclination. The adjacent teeth near the extraction site tipped into the space; hence, the extraction space decreased over time.

### Factors influencing physiologic drift

The monthly average velocities of the marked changes in variables were calculated (Table 2) and used to explore the influences of other factors, such as sex, age, Angle classification, drift duration and crowding prior to extraction, on physiologic drift.

**Table 2:** Average monthly velocity of the change in each variable.

Average velocity of change	N	Mean	SD	95% confidence interval		T value	p value
				Lower limit	Upper limit		
**Angulation (°/month)**							
V_U6a_	150	1.127	1.389	0.907	1.348	10.100	<0.01
V_U5a_	150	1.541	2.041	1.217	1.864	9.399	<0.01
V_U3a_	150	−1.268	1.966	−1.580	−0.956	−8.029	<0.01
**Inclination (°/month)**							
V_U6i_	150	−0.557	1.982	−0.872	−0.242	−3.501	<0.01
V_U3i_	150	−1.096	2.445	−1.484	−0.708	−5.581	<0.01
**Rotation (°/month)**							
V_U3r_	150	−1.083	2.067	−1.411	−0.755	−6.520	<0.01
**Mesiodistal displacement (mm/month)**							
V_U6m_	150	0.386	0.430	0.318	0.454	11.162	<0.01
V_U5m_	150	0.451	0.422	0.384	0.518	13.295	<0.01
V_U3m_	150	−0.482	0.586	−0.575	−0.389	10.241	<0.01
**Vertical displacement (mm/month)**							
V_U3v_	150	0.324	0.531	0.240	0.409	7.602	<0.01
**Extraction space closure (mm/month)**							
V_SC_	150	−0.792	0.621	−0.891	−0.396	−15.868	<0.01

Angulation: mesial tipping (+) or distal tipping (−).

Inclination: labioversion (+) or linguoversion (−).

Rotation: mesial inward rotation (+) or distal inward rotation (−).

Mesiodistal displacement: mesial movement (+) or distal movement (−).

Vertical displacement: extrusion (+) or intrusion (−).

Extraction space closure: space increase (+) or space decrease (−).

Multiple linear regression models (Table 3) showed that sex and the Angle classification have no significant influence on all the velocities. All the velocities decrease with drift time except V_U6i_ and V_U3r_. Age and crowding may have a moderate effect on certain velocities. V_U6a_, V_U6m_, V_U5a_, V_U5m_, and V_SC_ may decrease with age. More severe crowding prior to extraction may lead to an increase in V_U3a_, V_U3i_, V_U3m_, V_U3v_, and V_SC_. However, these factors may have a limited influence on the velocity of physiologic drift because the R² of all the linear regression models only ranged from 0.136 to 0.225.

**Table 3:** Multiple linear regression model of the velocity of each variable.

Velocity of variables	Standardized Coefficients							R²	F
	Sex	Age	Angle I	Angle II	Angle III	Crowding	Drift duration		
V_U6a_	—	−0.333*	REF	−0.037	0.086	—	−0.280*	0.136	0.001
V_U6i_	—	—	REF	0.029	−0.087	—	—	0.010	0.452
V_U6m_	—	−0.270*	REF	−0.046	0.066	0.037	−0.326*	0.156	0.035
V_U5a_	—	−0.284*	REF	−0.093	0.169	—	−0.303*	0.158	0.001
V_U5m_	—	−0.346*	REF	−0.033	0.073	—	−0.362*	0.172	<0.001
V_U3a_	—	—	REF	0.142	0.087	−0.209*	0.207*	0.171	0.013
V_U3i_	—	—	REF	−0.007	−0.078	−0.228*	0.234*	0.172	0.005
V_U3r_	—	0.048	REF	−0.032	−0.073	0.020	—	0.083	0.555
V_U3m_	—	—	REF	0.041	−0.089	−0.241*	0.275*	0.197	0.001
V_U3v_	—	—	REF	0.049	−0.051	0.205*	−0.262*	0.159	0.019
V_SC_	—	0.358*	REF	0.108	−0.104	−0.216*	0.294*	0.225	<0.001

^*^Standardized Coefficients, p value < 0.05

Angulation: mesial tipping (+) or distal tipping (−).

Inclination: labioversion (+) or linguoversion (−).

Rotation: mesial inward rotation (+) or distal inward rotation (−).

Mesiodistal displacement: mesial movement (+) or distal movement (−).

Vertical displacement: extrusion (+) or intrusion (−).

Extraction space closure: space increase (+) or space decrease (−).

## Discussion

Manual measurement, via lateral cephalometric radiographs and dental cast models, was used in most previous studies^28–33,35–37^ of dentition drift to assess tooth movement. However, only certain aspects of tooth movement were analyzed. Tipping and displacement of the teeth in the mesial-distal direction were often considered^28,31,37^. Closure of the extraction space was also discussed by some researchers^29,30,33^. Only a few studies focused on the change in other directions such as transversal angulations^32^ and rotation^37^. A reason for this may be because cephalometric measurement may only reflect the changes in the sagittal and vertical directions, and the manual measurement of pre- and post-extraction cast models could not assess the relative 3D changes accurately using 2 independent models. However, a rare 3D study of the physiologic drift^40^ of maxillary dentition also did not measure rotation and vertical changes. Most of the physiologic drift occurred in the first 6–12 months^29,30,32,37^, but the observation intervals were usually longer than this quick period of drift^28,30–33,37^, making it difficult to evaluate the continuous and specific characteristics of physiologic drift during this period. The use of a 3D laser scanner to digitalize the cast provides a new and convenient option to measure tooth displacement that is considered accurate and reliable^44–46^. Using 3D methods^47,48^ in this study, we conducted comprehensive and accurate measurements of 3D changes in the teeth after maxillary first premolar extractions at about 1-month interval.

Table 1 shows significant changes during the observation, and Table 2 shows the average velocity and direction. The most marked angular and linear change in the measured teeth exhibited a change towards the mesiodistal direction. These teeth showed marked movement and tipped into the extraction space, consistent with several other studies^28–32,35,37^. Additionally, the present study measured the changes in labiolingual inclination and rotation for each tooth using 3D methods. U6 and U3 showed lingual inclination, whereas U5 showed no significant inclination (Pearson’s r, p > 0.05). The difference in the drift pattern between the posterior teeth and canines may result from the lack of occlusal interference with the canines. U3 showed significant extrusion and rotation, although U6 and U5 did not, possibly because these teeth had more occlusal contact^16^ and were multirooted.

The findings from the present study and previous research have shown that tipping is the main style of movement during physiologic drift after extraction, and this phenomenon could partially prove the hypothesis that transseptal fibers, which are embedded in the cementum between adjacent teeth and extend interproximally over the alveolar bone crest, play an important role in physiologic drift. Kusters^49^ and Murphy^13^ found that transseptal fibers can self-repair and reconnect adjacent teeth after destruction of the periodontal ligament or extraction. Many previous studies^15–18,50^ also proposed that the reconstruction of transseptal fibers is the main cause of physiologic drift. There are some other potential causes for movement of the teeth into the extraction space. The position of the teeth may be influenced by the soft tissues surrounding them, and they remain stable in the equilibrium state of forces of the stomatognathic system^1,2,51,52^. Ogushi^52^ found that lip and cheek muscle pressure was highest at the canine. Hence, it is possible that, after extraction, the arch continuity may have been broken and the posterior support of canines was missed, leading the canines to move distally under the effect of the lip muscle. Moreover, Southard *et al*.^53,54^ measured an anterior component of occlusal force and reported its influence on dental alignment, which could also be a reason for the mesial drift of the upper molars after first premolar extraction.

Although this study of maxillary dentition indicated that the trend of physiologic drift of the maxillary teeth was similar to that of mandibular teeth, the speed of the drift was different. Panpadreas^31^ observed that mandibular dentition drifted approximately 10 months after lower first premolar extraction and found that the lower first molars had completely moved mesially by approximately 1.19 mm and had tipped forward by approximately 1.90°. Weber’s research^35^ showed that the lower first molars moved mesially by approximately 1.6 and 2.6 mm after the first and second premolar extraction, and the canines moved distally by approximately 4.4 mm over the 2.5 years. Additionally, Mamopoulou^37^ found that the lower first molar had moved forward by 2.5 mm at 1 year after second premolar extraction. Stephens^29^ observed that the extraction space decreased by approximately 1.6 mm in women and 3.23 mm in men during the first 6 months after the first premolar extraction. Swessi^32^ reported that the lower first molar did not markedly move during the long-term observation after the first premolar extraction. The present study found that the maxillary extraction space decreased by an average of 0.792 mm/month after extraction, leading to an approximately 4-mm decrease in the extraction space over 6 months. U6 also showed faster tipping (1.127°/month) and movement (0.386 mm/month) over the 6-month period (Table 2), which may be related to the looser nature of the alveolar bone of the maxilla, relative to the mandible^55,56^. Hence, the physiologic strength after tooth extraction makes it easier for the maxillary teeth to move.

In many animal experiments, researchers have used rats^21–25^ or *Macaca irus*^14–20^ to explore the reasons for physiologic drift. The marked differences in the biological nature between these animals and humans made it difficult to identify the true reason for human dental drift after extraction. In rats, there is a lifelong distal drift of the dentition^25^; in *Macaca irus*, the natural spaces are more sufficient than those in human dentition^20^. These findings may be valuable, but clinical studies on humans should be preferred to these animal experiments. To our knowledge, this is the first comprehensive 3D study of maxillary dental physiologic drift after premolar extraction that could help better understand the pattern of physiologic drift in the upper arch.

To explore the influences of several common factors on physiologic drift, multiple linear regression procedures were performed (Table 3). Because the significant variable changes (Table 1) were markedly correlated with drift time, the average velocity of each movement was calculated and chosen as the dependent variable to eliminate the marked effects of drift duration on the quantity of movement. The standardized coefficients between these factors and each velocity, as well as their significance, were also calculated. Sex had no influence on drift, a finding that was consistent with that of Gragg^33^, but differed from the research of Stephens on the mandible^29^. The influence of the Angle classification on physiologic drift has not been discussed, and it was not found to be significant in the present study.

As noted in Table 3, the factors significantly influencing physiologic drift included drift duration, age and crowding prior to extraction. Nearly all the velocities decreased with drift duration, indicating that physiologic drift may slow down over time. The extraction spaces tended to close faster in younger patients and crowding had a positive effect on the space closure. A younger sample was associated with faster movement and mesial tipping of U6 and U5, consistent with certain animal experiments^24,25^. One reason could be that most of the younger patients in the present study were in the late peak period of growth and development. Johnston’s research^34^ reported that excess growth of the mandible during this period would lead to mesial drift of the maxillary molars to establish a stable molar relationship for dentoalveolar compensation, which could explain why the upper molars of younger patients drifted mesially to a greater degree after premolar extraction. Moreover, the age-related effects on tissue reaction^57^, the proliferative rate of PDL cells^58^, osteoclastic activities^59^ and mineral density in alveolar tissues^60^ could explain the greater drift in the younger population.

Crowding prior to extraction had a positive effect on the velocity of canine movement in both the horizontal and vertical directions but had no effect on the posterior teeth, probably because crowding primarily occurred in the anterior teeth. Canine malposition is very common among orthodontic patients. The extraction of the first premolar could provide some spaces for these canines with malocclusion. Based on our findings (Table 3), more severe crowding could lead to faster distal tipping, distal displacement, and canine extrusion. Research on mandibular dentition^26,31^ reported a relief of crowding after extraction. In the present study, the distal direction of canine movement could help reduce crowding. Although the dynamic interaction between crowding and the physiologic drift of canines was complicated to evaluate, we chose crowding before extraction as an independent variable because it was convenient to measure.

Some other endogenous factors could affect the drift, such as periodontitis and eruption of a second molar. Kirschneck^61^ reported that experimental periodontitis in rats accelerated the tooth movement induced by orthodontic forces. The dental root resorption and periodontal bone loss were also increased during periodontitis, most likely caused by the upregulation of osteoclastic activity. Thus, the possibility that periodontitis could affect the physiologic drift should not be neglected. We excluded patients with moderate to severe gingivitis or periodontal disease and monitored the periodontal status of the participants to eliminate this impact as much as possible. Studies^62,63^ have shown that an erupting third molar could lead to crowding of the anterior teeth, indicating that an erupting molar could push the adjacent teeth forward. However, the influence of eruption of the second maxillary molar was not evaluated in the present study due to two reasons. First, the eruption of the second maxillary molar is correlated with age, which may lead to collinearity of the independent variables in the linear regression model. Second, it was difficult to evaluate the specific stage of the second molar eruption before extraction and at the end of observation for each sample.

Exogenous factors on the rate of tooth movement could also affect the drift, such as common prescription medication, smoking and alcohol abuse. Makrygiannakis’ systematic review^64^ concluded that commonly prescribed medications may exhibit variable effects on the rate of orthodontic tooth movement. For example, the rate of orthodontic tooth movement was shown to increase after the administration of diazepam or vitamin C but decrease after the administration of simvastatin, atorvastatin and calcium compounds. Although the quality of the available evidence was considered at best as low in this review, we still excluded patients with chronic or metabolic diseases, who usually need the long-term administration of prescription medication. Increased orthodontic tooth movement was found with regular nicotine intake in a rat model^65^. Moreover, the extent of root resorption, osteoclast activity, and gene expression of inflammatory and osteoclast markers were significantly increased under the influence of nicotine in this study, indicating that patients with smoking habits may present accelerated orthodontic tooth movement. By contrast, alcohol abuse may have an inhibitory effect on tooth movement during orthodontic therapy^66^. Araujo’s research^66^ showed that bone resorption at the end of tooth movement decreased after the administration of 20% ethanol, suggesting a delay in orthodontic tooth movement. We excluded patients with the above-mentioned life habits to eliminate such potential impacts on the physiologic drift.

Alexander^26^ suggested that bonding appliances to the lower arch should be delayed to allow for natural drifting to relieve anterior crowding after extraction, consistent with other studies^31,32,37^ on mandibular dentition. Compared with studies of the lower arch, the present study showed that the first molars had tipped and displaced faster and to a greater extent over a similar period (Fig. 1, Table 2), whereas the extraction space decrease showed a similar trend. Moreover, the quantity of displacement of the first molar and canine was similar (approximately 2 mm over 6 months, Fig. 1b). This result differed from that of studies on mandibular dentition, which showed that most extraction space closures were attributed to the distal drift of the canines^35,36^. Table 3 indicates that the younger patients exhibited faster mesial movement of the posterior teeth. In addition to the mesial drift of the upper molars caused by excess growth of the mandible (as dentoalveolar compensation)^34^, the upper first molars (perhaps the most important anchorage teeth) could have a higher risk of physiologic anchorage loss, relative to the lower ones after premolar extraction, particularly among younger orthodontic patients during the period of growth and development.

Molars can drift for at least 6 months after extraction (Fig. 1a,b), indicating that they can drift during the time between extraction and start of treatment, and even during the early stage of the treatment, usually using light forces applied by the soft wires for alignment. The effect of such weak forces on physiologic drift of the maxillary first molar remains unclear, but it is known that, for cases requiring maximum control of molar anchorage, this mesial drift of the molars should be controlled as soon as possible after premolar extraction.

For canines, distal tipping, distal movement, and extrusion are beneficial for alignment, particularly among cases with labially ectopic canines or those with anterior teeth crowding. Therefore, the physiologic drift of the canines can be used during treatment. An appliance technology can better offer the potential to control the mesial drift of the upper first molar and could help exploit the distal drift of the canines simultaneously. PASS technology^27^ considers both aspects. The XBT buccal tube could prevent the continuous mesial tipping movement of the upper first molar initially, and the low friction design at the canine brackets can minimize the prevention of the distal drift, thus achieving both objectives with one option.

Orthodontists usually decide to extract healthy premolars for patients with severe tooth discrepancy^8^ or crowding^11^. Additionally, extraction is a commonly used strategy to affect the soft tissue profile^10^ and skeletal vertical dimension^9^. However, according to some recent studies^67–69^, the effects on the skeletal and soft tissue of extraction orthodontic treatment could not be confirmed and might be overestimated. Kirschneck’s retrospective cohort study^67^ reported that premolar extraction in juvenile borderline patients did not lead to a significant reduction in the skeletal and vertical dimensions compared with non-extraction treatment in the short-term. Kouvelis’ systematic review^68^ also concluded that the extraction treatment protocol aiming to reduce or control the vertical dimension did not seem to be an evidence-based clinical approach. Both Kirschneck’s^67^ and Iared’s^69^ studies showed that differences between the changes in the patients’ facial profiles resulting from orthodontic treatment with and without extraction were clinically irrelevant or not significant. The duration of orthodontic treatment was significantly longer in patients who underwent extraction, and Iared^69^ suggested the cephalometric parameter, Li-E, could help decision making in borderline cases. Combined with the results in the present study that maxillary premolar extraction could lead to molar anchorage loss to some extent by inducing the mesial drift of the first molars, the decision on extraction for orthodontic reasons should be made more carefully. More individual factors and evidence-based implications should be considered when making an integrated treatment plan.

The general trends of the physiologic drift of the remaining adjacent teeth after first premolar extraction could be described in this cross-sectional study, but this study had some limitations. Errors may be caused due to cast preparation, cast digitalization and measurement^47,48^ at each interval because the 3D dental scanner (R700 linear laser scanner; 3shape Corp., Denmark) can register numerous coordinates in three dimensions with ±20 μm precision of the scan. The generalizability of these results might be limited by the characteristics of the study sample because this single-center study included mostly teenage and young adult patients seeking orthodontic treatment. There are some bias factors in the characteristics of the participants because most of the patients with long-term observation required mild or medium anchorage. Some of the observed results from this study showed high individual variation, consistent with some other research^31,32,37^, indicating that, although the general trends of the physiologic drift could be summarized, some individual differences exist in the drift of patients with different patterns of malocclusion. Some factors significantly influencing the physiologic drift were filtered by multiple linear regression procedures, while the R² of all the linear regression models only ranged from 0.136 to 0.225, indicating that these factors might only have limited contribution to the physiologic changes in these samples. Thus, the average velocities of these changes generally remained stable for at least 6 months after extraction in this study. However, the prolonged observation time would impede the therapy because the uncontrolled tooth movement during physiologic drift could lead to maxillary molar anchorage loss and affect the treatment outcomes. Although some possible biasing influences on the rate of tooth movement, such as prescription medication, smoking and alcohol abuse, were eliminated in the patient cohort, it was uncertain whether the participants started the administration of drugs, nicotine or alcohol during the drift. Additional factors^70^ may also influence physiologic drift, in addition to those considered in this study, such as occlusal influences, muscle pressure, drift of the lower dentition, and mandible growth. For patients with different patterns of malocclusion, it is difficult to control these factors and explore their influence on physiologic drift with this sample size. Hence, further research with a larger sample size and more comprehensive analysis is needed.

Despite these limitations, this study explored the continuous and specific three-dimensional characteristics of physiologic drift of the upper arch following tooth extraction and assessed some factors influencing the drift. We believed that it had certain clinical value in orthodontics and could fill the gaps of previous studies to some degree.

## Conclusion

All the measured teeth showed a similar tipping movement toward the extraction spaces, leading to a decrease of the extraction spaces. The maxillary first molars and canines tended to move into the spaces at a similar speed, indicating that the mesial drift of molars after premolar extraction may cause molar anchorage loss to some degree and should be prevented as soon as possible after premolar extraction in cases in which maximum control of molar anchorage is needed. Moreover, the pattern of physiologic drift of maxillary canines after premolar extraction helps relieve crowding and labially ectopic canine alignment. Additionally, drift time, age and crowding can influence physiologic drift. Physiologic drift tended to slow over time. In younger patients, posterior teeth tended to drift mesially at a faster rate and the extraction spaces tended to close faster. Crowding may have a positive effect on the drift of canines and space closure, but sex and the angle classification did not significantly affect drift.

## Methods

### Research design and selection of participants

This study was designed to explore the 3D characteristics of physiologic drift of the upper arch following maxillary first premolar extraction and associated factors. To gain as many generalizable results as possible, we planned to evaluate teenage and young adult (Under 30) patients, who made up the largest group of patients seeking orthodontic treatment in our department. For these patients, the decisions on bilateral maxillary first premolar extraction were made by senior orthodontists. To reduce the possible bias, the patients were enrolled based on the following inclusion criteria: (1) permanent dentition, (2) no molar crossbite or buccal crossbite, (3) no orthodontic history, (4) no congenital missing teeth, and (5) a complete crown. The exclusion criteria were as follows: (1) systemic disease, including chronic or metabolic diseases requiring the long-term administration of prescription medication, (2) congenital craniofacial deformity and syndromes, including cleft lip and palate, (3) maxillary surgical history or trauma history, (4) impacted teeth, (5) moderate to severe gingivitis or periodontal disease, (6) periapical periodontitis of the posterior teeth, and (7) smoking or alcohol abuse.

**Figure 2:**
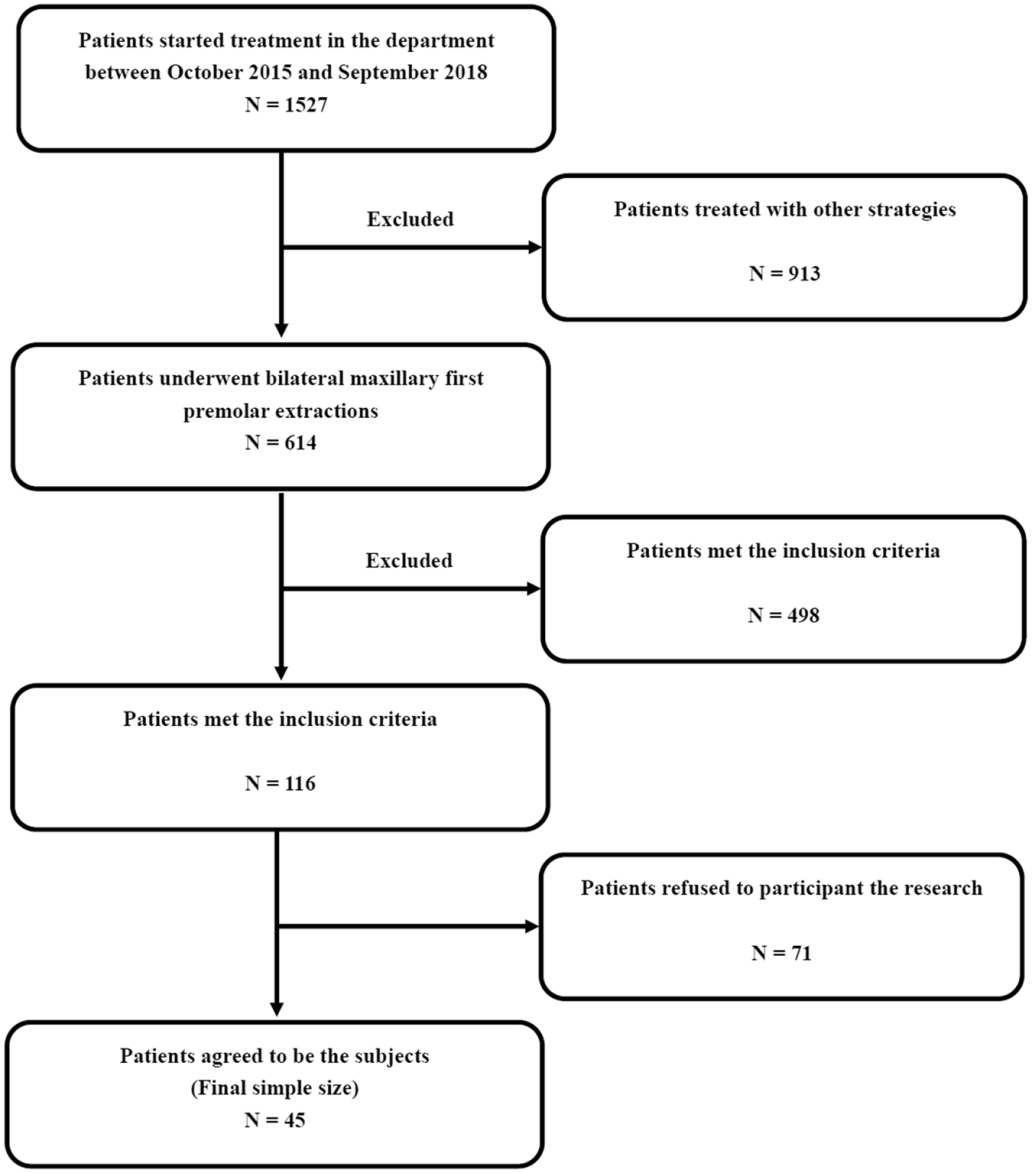
Flow chart detailing the patient selection process.

There were 1,527 patients in total who started treatment in our department between October 2015 and September 2018. Six hundred fourteen patients underwent bilateral maxillary first premolar extraction, and 116 of them met the inclusion criteria. The observation time was from the extraction sites generated to the day for fixed appliance bonding. Different drift durations were determined for each patient, ensuring that their treatment was not affected. Because the observation could probably extend the period of treatment, finally 45 patients (Asians, 10 male and 35 female) agreed to be the subjects (Fig. 2). As a preliminary study, there were no relevant data to refer to when deciding the sample size. In some relevant research, the sample size was 13 to 50^28–32,35,37,40^, while no researcher explained how the study size was determined. To obtain as much data as possible, we decided to enroll all the available patients. This study was approved by the institutional review board of Peking University School of Stomatology (PKUSSIRB-201310072). Written informed consent was obtained from all the subjects before the study. The methods were carried out in accordance with the relevant guidelines and regulations.

### Data acquisition

For each patient, the pre-treatment cast (T0) was obtained prior to extraction and the inter-observation casts (Tx) were obtained after extraction and before fixed appliance bonding. The dental and periodontal status of each patient was monitored by dentists during the observation. Next, 120 dental casts (45 casts at T0 and 75 casts at Tx) were obtained and scanned with a 3D dental scanner (R700 linear laser scanner; 3shape Corp., Denmark) and were virtually constructed into digital casts using the Rapidform 2006 program (Inus Technology, Korea). Crowding prior to extraction in each patient was measured using Proffit’s method^71^ and was recorded in millimeters.

### Three-dimensional measurement

**Figure 3:**
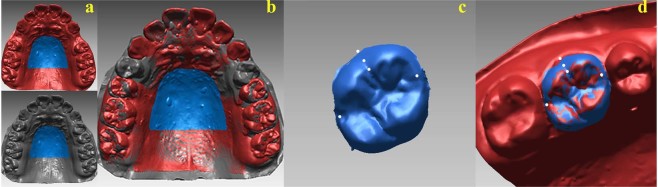
Three-dimensional superimposition method used in this research. (**a**) Medial 2/3 of the palatal region between the third rugae and line in contact with the distal surface of the bilateral maxillary first molars. (**b**) Two digital casts at different periods (gray and red) superimposed at the stable palatal region^47^. (**c**) The marked points are bonded to the copied crown. (**d**) The digital shell of the target tooth (blue) was transferred to the digital cast of the next period and was well-matched with the identified tooth (red).

**Figure 4:**
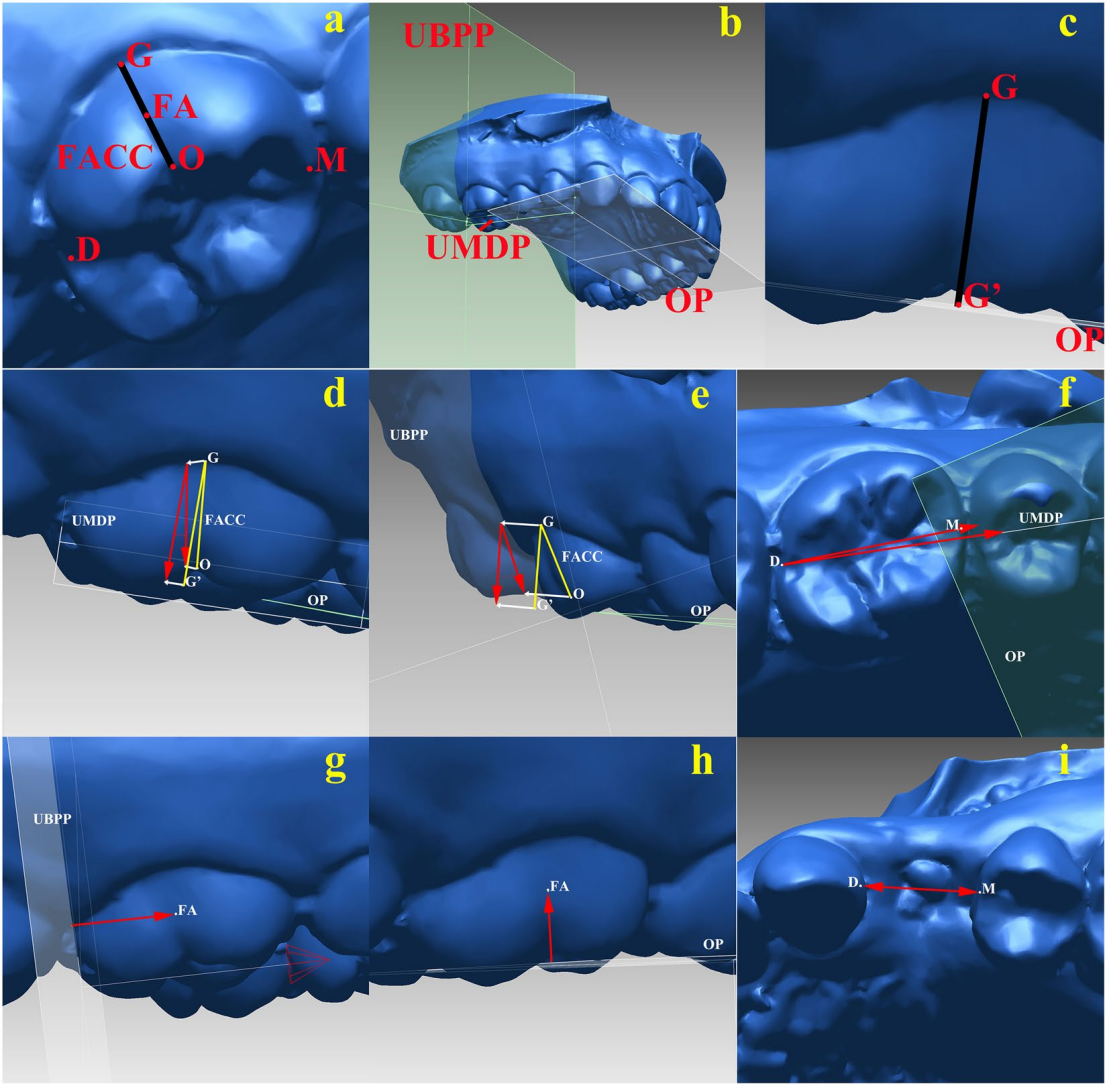
Measurements of the tooth 3D information. (**a**–**c**) Reference points, lines, and planes defined on a digital cast: (**d**) angulation; (**e**) inclination; (**f**) rotation; (**g**) mesiodistal displacement; (**h**) vertical displacement; (**i**) extraction space closure.

The T0 and Tx digital casts were superimposed via stable palatal region superimposition^47^ (Fig. 3a,b). Because bilateral premolar extraction was not conducted at the same time, the tooth displacement and drift duration on both sides were analyzed separately (thus, there were 150 extraction sides at Tx, and the average drift duration was 81.66 ± 70.03 days). Unilateral crowding prior to extraction (from the most mesial point of incisor to the most distal point of the first molar) was measured^71^. The definitions of the reference points, lines, and planes (some were based on Cho *et al*.^48^) (Fig. 4, Table 4) were set for measurement. The coordinate systems of the digital casts in different periods were unified based on the digital casts at T0. In cases with fixed-point error, the fixed reference point of each tooth was transferred to the identified tooth on the other digital cast during the next period (Fig. 3c,d). The 3D tooth measurement data, including tooth angulation, inclination, rotation, mesiodistal displacement, vertical displacement, and extraction space closure, were measured for each cast after superimposition (Fig. 4, Table 4). The variables were measured 3 times by 1 examiner over a 2-week interval. Changes in all the above-mentioned variables between each Tx and the corresponding T0 (Tx − T0) were recorded as the raw data. The directions of the changes were as follows: angulation: mesial tipping (+) or distal tipping (−); inclination: labioversion (+) or linguoversion (−); rotation: mesial inward rotation (+) or distal inward rotation (−); mesiodistal displacement: mesial movement (+) or distal movement (−); vertical displacement: extrusion (+) or intrusion (−); extraction space closure: space increase (+) or space decrease (−).

**Table 4:** Definitions and abbreviations of the reference point/line/plane and measurements of tooth movement.

	Definition
**Reference point/line/plane**	
Gingival point (G)	the lowest and most concave point in the gingival margin of the clinical crown
Occlusal point (O)	the cusp tip of the canine and premolar; the most concave point of the buccal marginal ridge of the molar
Mesial point (M)	the most mesial point of the central fissure on the occlusal surface; for canines, this is the mesial end of the segment, representing the maximum mesiodistal dimension from the occlusal view
Distal point (D)	the most distal point of the central fissure on the occlusal surface; for canines, this is the distal end of the segment, representing the maximum mesiodistal dimension from the occlusal view
Facial axis (FACC)	the facial axis of the clinical crown; the segment connecting G and O
FA point (FA)	midpoint of the FACC
Occlusal plane* (OP)	the plane established by the midpoints of the right and left incisal edges and mesiobuccal cusp tips of the right and left first molars
Unilateral posterior mesiodistal plane* (UMDP)	the plane passing the foot of M of the first premolar and D of the first molar at the OP, perpendicular to the OP; the M of the second premolar is used if the first premolar is severely dislocated
Unilateral posterior buccopalatal plane* (UBPP)	the plane passing the foot of D of the first molar at the OP, perpendicular to the OP and UMDP
G’ point (G’)	the projection of G on the OP
**Measurements of tooth movement (T**_**X**_ **−** **T**_**0**_)**	
Angulation (U6a, U5a, U3a)	the projection of the angle between FACC and GG’ on UMDP
Inclination (U6i, U5i, U3i)	the projection of the angle between FACC and GG’ on UBPP
Rotation (U6r, U5r, U3r)	the angle between the projection of MD on OP and UMDP
Mesiodistal displacement (U6m, U5m, U3m)	the distance between FA and UMDP
Vertical displacement (U6v, U5v, U3v)	the distance between FA and UBPP
Extraction space closure (SC)	the distance between D of the canine and M of the second premolar

*All reference planes were set based on the pre-treatment dental casts.

**U6 for first molar, U5 for second premolar, U3 for canine.

### Statistical analysis and methods

Intraclass correlation coefficients (ICC) for the measurements of the angular variable, linear variable, and space closure were computed to assess intra-examiner reliability (repeatability). The results were 0.94, 0.91, and 0.95 for measurements of the angles, the linear distances, and extraction space closure, respectively. Because the assessment showed an excellent ICC value^72^, the first measurement was used.

The characteristics of the 150 extraction sites at T_X_ were summarized based on descriptive statistics, and the data were used for further analyses.

First, the Pearson’s correlation coefficients between all the changes in the variables and durations of drift (days) were calculated to test the significance of the change over time. Next, the markedly changed variables were selected for further analyses.

Changes in the selected variables between Tx and T0 were grouped based on the months (30 days as a month, rounded up to the nearest number) of drift at Tx. Thus, they were divided into 7 groups, including 1-month to 6-month groups, and the over 6-month group. The average significant changes in each month was calculated to create a line chart to directly observe the amount and tendency of drift.

The average velocity of movement over 1 month (V_X_ = S_X_*30/T; V: average velocity; S: change in the angular or linear variable; T: days between extraction and observation, 30 days as a month; X: variable of tooth movement) was calculated. Two-tailed single-sample t-test was used to test the significance of the velocity (Test Value: 0), and the 95% confidence interval was calculated.

Multiple linear regression was used to explore the influences of different factors including sex,age, angle classification, crowding, and drift duration on the velocity of the physiologic drift. The average velocity was chosen as the dependent variable and certain other factors were chosen as the independent variables. For each velocity, the angle classification (a categorical variable) was set as a dummy variable and was entered first. A forward stepwise linear regression procedure was then performed for the other factors.

The results of the Kolmogorov–Smirnov test showed that all the data were approximately normally distributed. Statistical significance was determined at the 1% and 5% levels of confidence. We used IBM SPSS Statistics 22 (IBM, Inc., Armonk, NY, USA) for statistical analyses.

## Data Availability

The datasets generated and analyzed during the current study are available from the corresponding author on reasonable request.
